# Placenta-Derived Secretions Promote Liver Dysfunction, and Hepatic Serum Amyloid A Mediates Kidney Inflammatory Response in a Preeclampsia-like Mouse Model

**DOI:** 10.3390/ijms262110737

**Published:** 2025-11-04

**Authors:** Ren Ozawa, Sae Suzuki, Ayaka Shirota, Shota Nomura, Takanori Komada, Masafumi Takahashi, Hisataka Iwata, Koumei Shirasuna

**Affiliations:** 1 Laboratory of Animal Reproduction, Department of Animal Science, Tokyo University of Agriculture, Atsugi, 243-0034, Japanh1iwata@nodai.ac.jp (H.I.); 2 Division of Inflammation Research, Center for Molecular Medicine, Jichi Medical University, Shimotsuke, 329-0498, Japan; t_komada@jichi.ac.jp (T.K.); masafumi2@jichi.ac.jp (M.T.)

**Keywords:** preeclampsia, serum amyloid A, organ communication, placenta

## Abstract

Preeclampsia (PE) is characterized by maternal hypertension accompanied with multi-organ dysfunction, such as maternal hepatic and renal dysfunction. Abnormal placental conditions may play a key role in regulating maternal organ function by promoting systemic inflammation. This study aimed to test the hypothesis that placenta-derived secretions contribute to hepatic and renal injury through interorgan communication using a PE-like mouse model. Pregnant mice were infused with angiotensin II (Ang II) from gestational day (GD) 12 (GD1 defined as the day of plug detection). Ang II infusion induced maternal hypertension, as well as liver injury (elevated serum amyloid A [SAA] secretion and alanine aminotransferase levels) and kidney injury (tubular damage with KIM-1 protein expression and immune cell infiltration). Treatment with placental-conditioned medium (CM) from Ang II-infused mice, but not from the control mice, stimulated SAA expression in liver cells. On the other hand, the effects of placental-CM from both the control and Ang II groups on kidney tubular cells were comparable. These findings suggest that placenta-derived secretions in the Ang II-induced PE-like phenotype specifically promote excessive SAA production in the liver. Furthermore, SAA administration in pregnant mice did not cause tubular injury but did promote renal immune cell infiltration, indicating that elevated hepatic SAA levels may contribute to maternal kidney inflammation. Taken together, these results suggest the presence of an in vivo organ network involving the placenta, liver, and kidneys during pregnancy, where dysfunction in one organ may exacerbate the pathogenesis of PE.

## 1. Introduction

Preeclampsia (PE) occurs in approximately 5% of all pregnancies and is characterized by maternal hypertension, proteinuria, fetal growth restriction, and multi-organ dysfunction, such as maternal hepatic and renal dysfunction [1]. Elevated concentrations of alanine aminotransferase (ALT), aspartate aminotransferase, and serum amyloid A (SAA) are detected in the circulation in patients with PE, indicating hepatic inflammation and the activation of acute-phase responses [2]. Proteinuria is a diagnostic hallmark of PE pathology, in which the levels of renal injury markers and inflammatory cytokines are elevated [3]. Systemic inflammation plays a key role in development of PE pathogenesis; thus, the major risk factors for PE are involved in chronic inflammatory disease, such as chronic hypertension, obesity, diabetes mellitus, and advanced maternal age [4]. Despite the considerable maternal and fetal risks associated with PE, its underlying pathogenesis remains incompletely understood.

The pathogenesis of PE is generally attributed to abnormal placental development, particularly insufficient spiral artery remodeling, which leads to placental ischemia and chronic inflammation. Placental tissue releases various factors into the maternal circulation, including anti-angiogenic proteins such as soluble fms-like tyrosine kinase-1 (sFlt-1), inflammatory cytokines, and extracellular vesicles [5,6,7]. sFlt-1 plays a central role in PE pathogenesis, impairs endothelial function, and contributes to hepatic and renal injury by sequestering the vascular endothelial growth factor [8,9,10]. Inflammatory cytokines and extracellular vesicles also impair maternal endothelial function and contribute to systemic inflammation, leading to multi-organ dysfunction. These placental-derived signals are thought to contribute to maternal organ dysfunction, yet the mechanisms by which they induce specific pathological changes in each organ remain unclear.

Serum amyloid A is an acute-phase protein predominantly produced by the liver in response to systemic inflammation [11]. Circulating SAA levels are elevated in patients with PE, particularly in patients with early-onset PE [12]. While SAA is typically induced by infectious or inflammatory stimuli, the mechanisms underlying its upregulation in PE remain poorly understood. Importantly, SAA is not merely a biomarker of inflammation but also acts as a pro-inflammatory mediator by stimulating cytokine and chemokine production and promoting immune cell activation [11]. Thus, elevated SAA in a PE situation may both reflect the maternal inflammatory state and have a potential role in further exacerbating organ dysfunction.

The aim of this study was to test the hypothesis that placenta-derived secretions contribute to hepatic and renal injury through an interorgan communication system underlying the pathogenesis of PE. In addition, we investigated whether liver-derived SAA contributes to development of PE pathogenesis and renal injury during pregnancy using a mouse model.

## 2. Results

### 2.1. Effect of Angiotensin II (Ang II) on Blood Pressure and Fetal Development in Pregnant Mice

**Figure 1:**
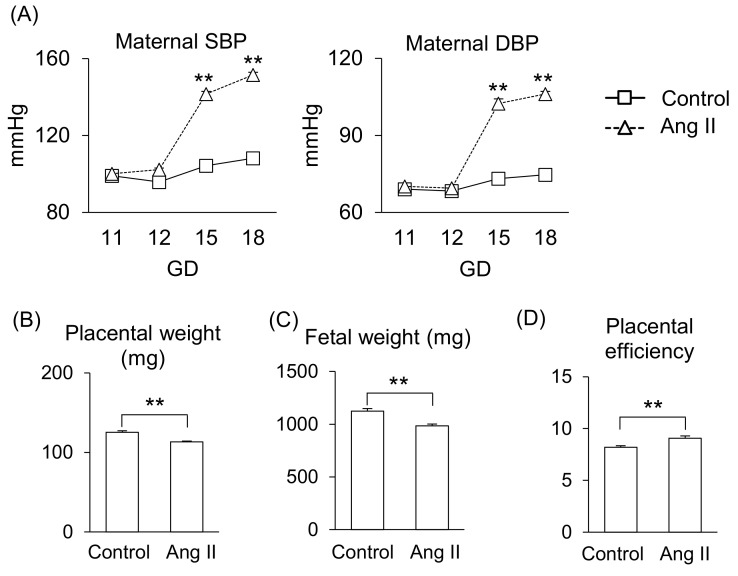
Effects of Ang II on maternal responses during pregnancy. Pregnant mice were infused with Ang II on GD12. (**A**) Maternal SBP and DBP. (**B**,**C**) Placental and fetal weights on GD18. (**D**) Placental efficiency was calculated by dividing fetal weight by placental weight. Data are expressed as mean ± SEM. ** *p* < 0.01.

First, we determined the effect of Ang II during pregnancy in mice. Ang II infusion on gestational day (GD) 12 significantly increased systolic blood pressure (SBP) and diastolic blood pressure (DPB) compared to that of the control group (Figure 1A), consistent with our previous report [13]. Also, Ang II infusion significantly reduced placental weight and fetal weight (Figure 1B,C), resulting in a shift in the balance of placental efficiency (Figure 1D).

### 2.2. Effect of Ang II on Maternal Liver in Pregnant Mice

**Figure 2:**
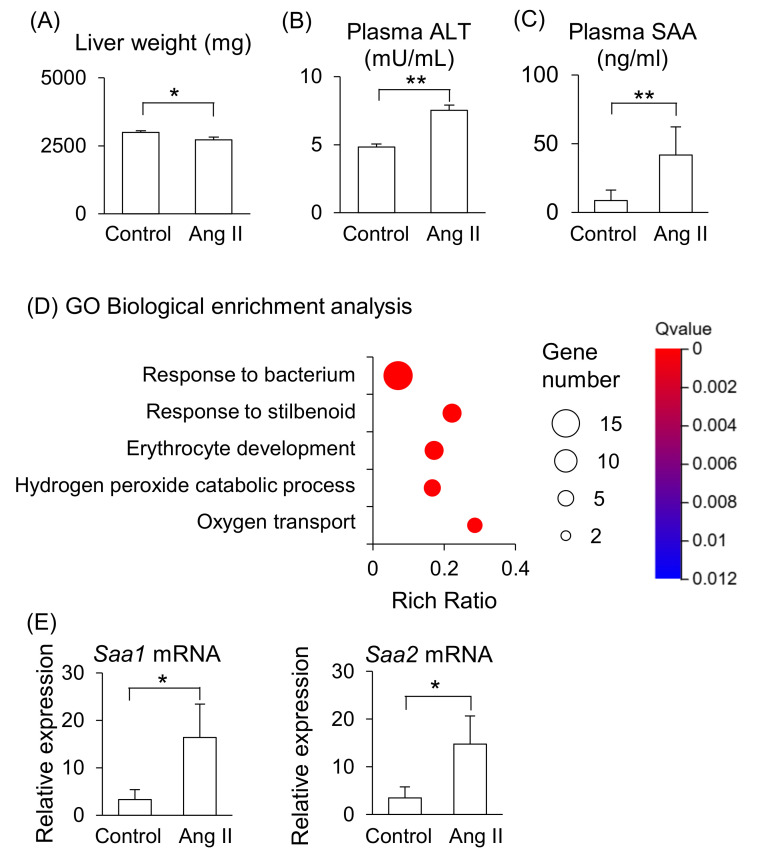
Effects of Ang II on maternal liver in pregnant mice. Pregnant mice were infused with Ang II on GD12. (**A**) Liver weight on GD18. (**B**,**C**) ALT and SAA levels in peripheral blood plasma on GD18. (**D**) GO biological enrichment analysis of differentially regulated genes in the liver between the control and Ang II; the top five pathways are displayed. (**E**) mRNA levels of *Saa1* and *Saa2* in the maternal liver were analyzed by RT-qPCR. Data are expressed as mean ± SEM. * *p* < 0.05 or ** *p* < 0.01.

Maternal liver weight was significantly lower in Ang II-infused mice (Figure 2A), and plasma levels of ALT and SAA were significantly higher in Ang II-infused mice than in the control mice (Figure 2B,C). RNA-seq was performed to further analyze the changes in liver function induced by Ang II infusion. The gene ontology (GO) biological enrichment analysis showed that most upregulated pathways were related to “Response to bacterium” in the Ang II-infused mice (Figure 2D). The fold changes *Hba-a2*, *Saa2*, *Lcn2*, and *Saa1* were higher in Ang II-infused mice than in the control mice among the upregulated genes (Supplemental Table S1). *Hba-a2* encodes hemoglobin α, adult chain 2, which functions as an antioxidant and is upregulated in liver dysfunction [14]. *Saa1* and *Saa2* encode acute-phase proteins that are primarily synthesized in the liver during inflammation. *Lcn2* encodes lipocalin-2, also known as neutrophil gelatinase-associated lipocalin, which is involved in iron regulation and responses to tissue injury [15]. These genes are associated with liver disease [11,14,16,17], indicating that Ang II infusion induced liver dysfunction and inflammation in the pregnant mice. We validated gene expression of *Saa* by RT-qPCR and confirmed that the mRNA levels of *Saa1* and *Saa2* were higher in the liver of Ang II-infused mice than in the control mice (Figure 2E). The mRNA expression levels of *Saa1* and *Saa2* positively correlated with the plasma SAA concentrations in the control and Ang II-infused mice (Supplemental Figure S1A,B). On the other hand, the fold change of *Dmbt1* (deleted in malignant brain tumors 1) was the lowest in Ang II–infused mice compared with control mice (Supplemental Table S1). DMBT1 is generally associated with the mucosal defense system and epithelial differentiation [18], and it is also involved in liver regenerative capacity, as well as cell fate determination and differentiation in the liver [19].

These results suggest that the maternal liver is the primary source of elevated circulating SAA in Ang II-infused pregnant mice, reflecting pro-inflammatory responses in the liver in this model.

### 2.3. Effect of Ang II on Maternal Kidneys in Pregnant Mice

**Figure 3:**
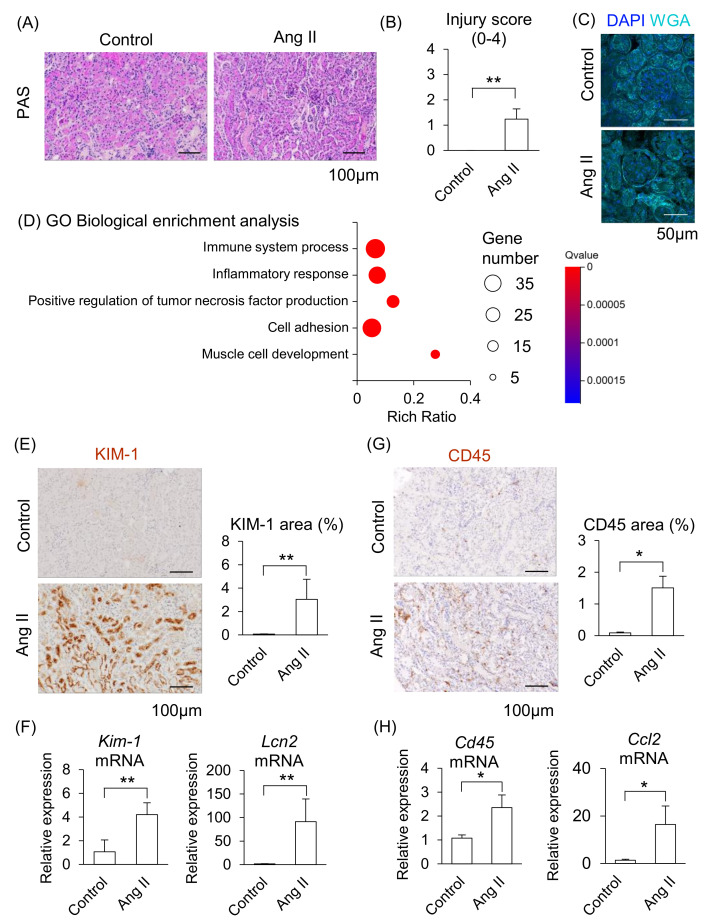
Effect of Ang II on maternal kidneys in pregnant mice. Pregnant mice were infused with Ang II on GD12. (**A**) Representative image of periodic acid-Schiff (PAS)-staining in the kidneys. (**B**) Tubular injury score of PAS staining samples of kidneys. (**C**) Representative image of glomerulus stained by WGA and DAPI. (**D**) GO biological enrichment analysis of differentially regulated genes in the kidneys between the control and Ang II; the top five pathways are displayed. (**E**) Representative images of immunohistochemistry for the KIM-1 are shown, and KIM-1-positive areas were quantified. (**F**) mRNA levels of *Kim-1* and *Lcn2* in the maternal kidneys. (**G**) Representative image of immunohistochemistry for the CD45 are shown, and CD45-positive areas were quantified. (**H**) mRNA levels of *Cd45* and *Ccl2* in the maternal kidneys were analyzed by RT-qPCR. Data are expressed as mean ± SEM. * *p* < 0.05 or ** *p* < 0.01.

We next determined whether maternal kidney injury occurred in Ang II-infused pregnant mice. In a histological analysis, distinct tubular damage was observed in the kidneys in Ang II-infused mice (Figure 3A), and renal injury scores were significantly elevated in Ang II-infused mice compared to the control mice (Figure 3B). Glomerular histological abnormalities were not detected in either the control or the Ang II groups (Figure 3C). These data indicate that Ang II led to apparent tubular injury but caused morphologically negligible alterations in the glomeruli of maternal kidneys.

We further assessed the changes in kidney function induced by Ang II infusion using RNA-seq. The GO biological enrichment analysis showed that most upregulated pathways related to “Immune system process” in the Ang II-infused mice compared with the control, followed by upregulated “Inflammatory response” (Figure 3D; detail changed genes are listed in Supplemental Tables S2 and S3). Among the upregulated genes, the fold changes of *Cd84*, *Il1f6*, *Lcn2*, *Clec4n*, and *Lilrb4a* were higher in Ang II-infused mice than in the control mice (Supplemental Table S2). CD84 is a membrane surface receptor expressed on immune cells that regulates cytokine release in macrophages [20]. IL1F6 is a member of the IL-1 cytokine family and is associated with renal inflammation [21]. CLEC4N belongs to a group of pattern recognition receptors in immune cells and modulates both innate and adaptive immune responses [22]. LILRB4A is a member of the leukocyte immunoglobulin-like receptor family and inhibits T-cell activity [23]. In addition, kidney injury markers, such as *S100a6*, *Lcn2*, and *Havcr1* (*Kim-1*), were included among the upregulated DEGs in Ang II-infused mice (Supplemental Table S4). S100A6 is a member of the S100 protein family and is involved in cell proliferation, differentiation, and cellular stress responses [24]. *Havcr1* (also known as *Kim-1*) encodes a type I membrane protein that serves as a marker of kidney injury and enhances the phagocytic function of tubular cells [25,26].

The area positively stained with KIM-1 was significantly larger in the renal tubules, and mRNA levels of *Kim-1* and *Lcn2* significantly increased in the kidneys of Ang II-infused mice compared to the control group (Figure 3E,F). Moreover, the area of CD45-positive immune cells was significantly larger, and mRNA levels of *Cd45* and *Ccl2* (major chemoattractant) were significantly increased in the kidneys of Ang II-infused mice compared to the control mice (Figure 3G,H). These results suggest that maternal kidney injury is associated with immune cell accumulation in Ang II-infused pregnant mice.

### 2.4. Effect of Placenta-Derived Conditioned Medium (CM) on Liver and Kidney Cells

**Figure 4:**
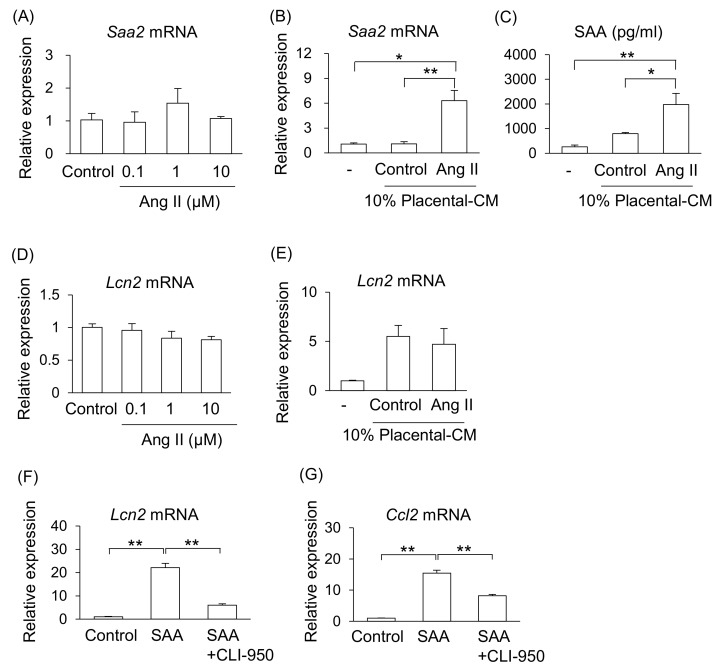
Effect of placenta-derived CM on liver and kidney cells. Mouse liver cell line (NCTC clone 1469) and kidney tubular cell line (TKC2) were treated with placenta-derived CM for 24 h. (**A**,**B**) mRNA expression of *Saa2* in liver cells was analyzed by RT-qPCR. (**C**) SAA secretion levels in the cultured supernatant were determined by ELISA. (**D**–**F**) mRNA expression of *Lcn2* in tubular cells was analyzed by RT-qPCR. (**G**) mRNA expressions of *Ccl2* in the tubular cells were analyzed by RT-qPCR. Data are expressed as mean ± SEM. * *p* < 0.05 or ** *p* < 0.01.

We investigated the possible mechanism that increase levels of SAA in Ang II-infused pregnant mice. One of the main sources of SAA is the liver; therefore, we examined the effect of Ang II on liver cells (NCTC clone 1469). However, treatment with Ang II did not affect mRNA expression of *Saa2* in liver cells (Figure 4A). As shown in Figure 1, Ang II adversely affected the placental tissue. Therefore, we hypothesized that placental abnormalities may adversely affect maternal organs via their secretions. Treatment with placental-CM from Ang II-infused pregnant mice, but not that from the control pregnant mice, significantly stimulated mRNA expression of *Saa2* and increased the amount of SAA secretion from liver cells (Figure 4B,C). Therefore, it is suggested that placental secretions changed by Ang II infusion, but not direct action of Ang II, may stimulate SAA production from the liver in pregnant mice.

Similarly, treatment with Ang II did not affect mRNA expression of *Lcn2* in kidney tubular cells (TKC2) (Figure 4D). Therefore, we examined whether placental abnormalities affect maternal kidney injury via their secretions. Although treatment with placental-CM upregulated the *Lcn2* mRNA expression levels in kidney tubular cells in both groups of mice, the reactivity did not differ between the control and Ang II-treated groups (Figure 4E). Thus, it is thought that placental secretions affected by Ang II infusion do not directly regulate *Lcn2* expression in kidney tubular cells. Therefore, it is suggested that although there is an organ connection between the placenta and kidney, there are other factors that can induce kidney injury.

As neither placental-CM nor Ang II affected kidney tubular cells, we focused on function of SAA on the kidneys. As a result, treatment with SAA significantly increased the mRNA expression of *Lcn2* and *Ccl2* on kidney tubular cells (Figure 4F,G). In addition, treatment with toll-like receptor 4 (TLR4) inhibitor (CLI-950) significantly suppressed these SAA effects (Figure 4F,G).

Therefore, these results hypothesized that (1) the composition of placental secretions changes under preeclamptic conditions, promoting SAA production from hepatocytes, and (2) the elevated SAA in PE condition may reach the kidneys systemically and contribute to renal tubular injury.

### 2.5. Effect of SAA on Kidneys in Pregnant Mice

**Figure 5:**
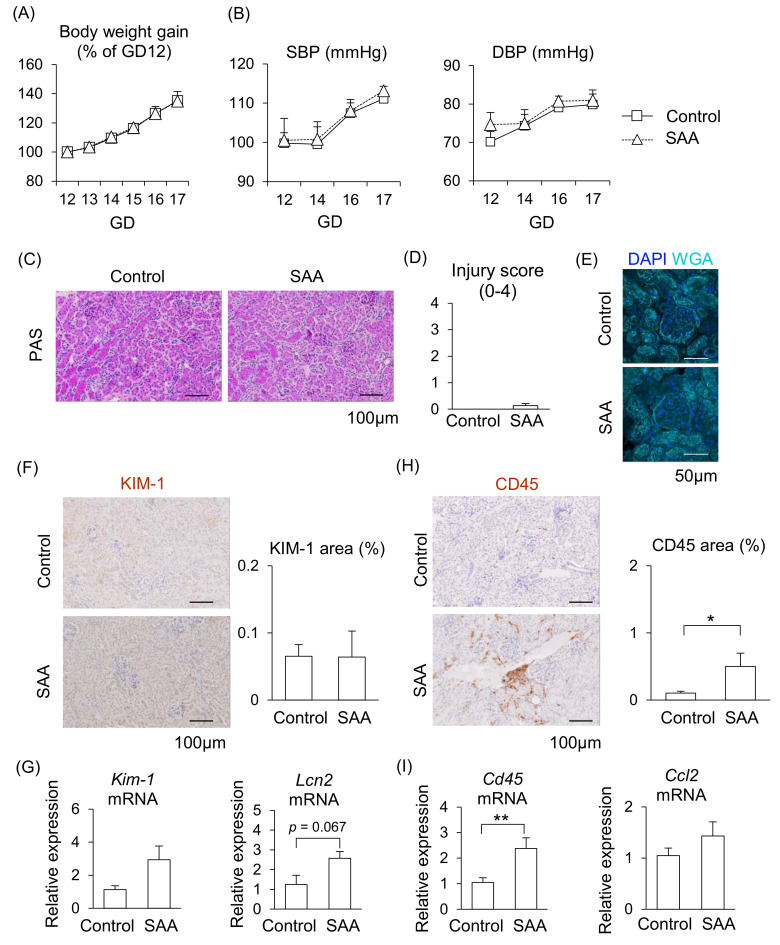
Effect of SAA on kidneys in pregnant mice. Pregnant mice were injected with SAA on GD12-16. (**A**) Maternal body weight gain during pregnancy. (**B**) Maternal blood pressure (SBP and DBP) were measured during pregnancy. (**C**) Representative image of the kidney with PAS staining. (**D**) Tubular injury score was examined using PAS staining. (**E**) Representative image of glomerulus stained by WGA and DAPI. (**F**) Representative images of immunohistochemistry for the KIM-1 were shown, and KIM-1-positive areas were quantified. (**G**) mRNA levels of *Kim-1* and *Lcn2* in the maternal kidneys. (**H**) Representative images of immunohistochemistry for CD45 are shown, and CD45-positive areas were quantified. (**I**) mRNA levels of *Cd45* and *Ccl2* in the kidneys. Data are expressed as mean ± SEM. * *p* < 0.05 or ** *p* < 0.01.

From the above findings, we hypothesized that higher levels of SAA contribute to PE pathogenesis; therefore, we investigated the direct effect of SAA in pregnant mice. During SAA administration in pregnant mice, although placenta weight increased, maternal body weight gain, blood pressure (both of SBP and DBP), fetal weight, and placental efficiency did not differ from the control mice (Figure 5A,B, Supplemental Figure S2), suggesting SAA alone does not contribute to the abnormal pregnancy.

We then examined whether SAA induces kidney injury in pregnant mice. Renal injury was not observed in the glomeruli or renal tubules after injecting SAA into the mice (Figure 5C–E). Although the mRNA expression levels of *Kim-1* and *Lcn2* tended to be higher following SAA administration, almost no expression of KIM-1 protein was observed in the renal tubules (Figure 5F,G). In contrast, the area of CD45-positive immune cells with *Cd45* mRNA expression were significantly larger in the kidneys of the SAA-injected mice compared to the control mice (Figure 5H,I). The mRNA expression levels of *Ccl2* did not differ between the SAA-injected mice and the control mice (Figure 5I). These findings suggest that higher SAA directly affects kidney function, but this effect alone is insufficient to damage the kidneys in pregnant mice.

## 3. Discussion

In the present study, we demonstrated that Ang II infusion induced PE-like symptoms with liver and kidney injury in pregnant mice. Our results suggest that placental secretions promote hepatic SAA production in pregnant mice. Moreover, higher SAA may contribute to promoting renal inflammatory responses in pregnant mice. These findings suggest that organ communication between placenta, liver, and kidneys, potentially mediated by placental secretions and hepatic SAA, is involved in the pathogenesis of PE in pregnant mice.

The circulating SAA levels in patients with PE, especially those with early-onset PE, are significantly increased compared with healthy pregnant women [12]. We focused on the liver as the main organ that produces SAA in Ang II-infused pregnant mice, and showed higher secretion and production of SAA with ALT elevation in the Ang II-infused group in the present study. Hepatic SAA production increased after infusing Ang II into ApoE-deficient mice [27], and hepatic SAA was suppressed by treatment with Ang II type 1 receptor blocker [28]. These findings suggest that Ang II infusion during pregnancy is associated with the induction of SAA production and secretion from the liver, resulting in liver dysfunction observed in pathology of PE.

Although Ang II infusion changed the function of the liver during pregnancy in mice, Ang II itself did not directly increase *Saa2* expression in hepatic cell line in this study. On the other hand, placental-CM of Ang II-infused mice increased the SAA production from liver cells. These results suggest that the placenta and liver are connected and that placenta-derived factors in PE pathological conditions induce inflammatory responses in the liver. Zhan et al. [29] suggested that Ang II infusion stimulates an interleukin (IL)-6-dependent increase in the hepatic SAA expression. In hepatocyte cell line (HepG2), IL-6 stimulation is essential for SAA expression and is enhanced by IL-1β and tumor necrosis factor-α [30,31]. We previously showed that Ang II infusion stimulated IL-6 production from placental tissue in mice [32]. Higher levels of IL-6 are also observed in patients with PE [6], suggesting that placenta-derived IL-6 may be a candidate for a mediator of liver dysfunction.

Other factors connecting placental and liver function include placenta-derived CD95L (FasL) [33]. CD95L levels were higher in serum in patients with HELLP syndrome, and CD95L induced apoptosis, which was suppressed by blocking CD95L in human hepatocytes [33]. Furthermore, intraperitoneal administration of mouse placental extract induced hepatic apoptosis, which was suppressed by inhibition of CD95L [33]. On the other hand, sFlt-1 overexpression induced liver damage in mice [34]. Therefore, we suggest that adverse condition of the placenta may increase the risk of maternal liver dysfunction during pregnancy, and further study is required to clarify the placental factors for induction of liver damage.

In addition to liver dysfunction, the major pathology in patients with PE is kidney dysfunction. The urinary and renal KIM-1 and Lcn2 levels are higher than normal in patients with severe PE [35,36]. Similarly, we showed that Ang II infusion induced renal tubular injury with the increase in KIM-1 expression and immune cell infiltration. Immune cells such as CD8^+^T cells, macrophages, and neutrophils are infiltrated in the kidneys and play key roles in inducing hypertension [37,38,39]. Our RNA-seq data showed that the individual markers of these immune cells such as *CD8a* (cytotoxic T cell marker), *Itgam* (also known *Cd11b*, neutrophil and macrophage marker), and *Lyz2* (lysozyme 2, macrophage marker) were increased by Ang II infusion in the kidneys. These findings suggest that Ang II infusion during pregnancy is associated with inducing kidney dysfunction and hypertension via activating the immune system observed in the pathology of PE.

During normal pregnancy, physiological changes occur in the kidneys to regulate blood volume for the mother and fetus and to maintain blood pressure. However, abnormal placentation in pregnancy-related hypertensive disorders, such as PE, leads to the release of antiangiogenic factors that can cause kidney injury, indicating the existence of placenta–kidney crosstalk during pregnancy [40]. Based on these findings, we next examined the effect of placental secretions on the kidneys using a similar approach as in the liver experiments. However, placental-CM of Ang II-infused pregnant mice did not stimulate mRNA expression of *Lcn2* in the kidney cells, unlike the liver cell experiment. Therefore, we hypothesized that placental-derived signals are weak in terms of causing tubular injury based on our experimental data obtained using placental-CM.

It has been reported that liver and renal dysfunction adversely affect each other, promoting distant organ damage through local inflammation [41]. Finally, we examined the effect of SAA from the liver as one of the factors inducing renal damage by Ang II infusion during pregnancy. Unfortunately, although SAA administration did not induce kidney injury as well as pregnancy complications, it increased immune cell infiltration in the kidneys, similar to the observation in Ang II infusion. Indeed, SAA regulates the production of chemokines in differential cell types [42] and promotes polarization toward M1 phenotypes in macrophages [43]. These findings suggest that hepatic SAA may act as a switch that initiates renal inflammation in PE-like conditions in mice.

In this study, we examined the effects of Ang II in a PE mouse model. In patients with PE, sensitivity to Ang II is enhanced despite reduced levels of circulating renin-angiotensin system components [44]. Moreover, transgenic mice overexpressing human renin and angiotensinogen exhibit elevated Ang II levels and develop PE-like pathology, including placental abnormalities [45]. These findings suggest that enhanced Ang II signaling contributes to the development of PE. Specifically, sFlt-1 has been identified as a factor that increases sensitivity to Ang II. Mice overexpressing sFlt-1 show heightened sensitivity to Ang II treatment, resulting in further elevation of blood pressure [46]. Since multiple mouse models can induce PE-like pathology other than Ang II, further research is required to determine whether organ interactions similar to those observed in this study also occur in other PE models.

The present study has some limitations. (1) What factor is secreted from the placenta in response to Ang II infusion remains unclear. The placenta can produce and release various types of signals, such as inflammatory cytokines, anti-angiogenic factors, hormones, and extracellular vesicles to the maternal circulation, and these factors can regulate maternal tissue function. Therefore, we plan to examine the potential role of placental-derived components under Ang II-induced PE-like conditions in mice. (2) SAA alone could only partially reproduce the pathology of PE-like symptoms in mice, so it is necessary to explore other candidate factors produced by the liver in association with the placenta and to examine the effects of these substances in combination on the kidneys during pregnancy.

In conclusion, the placenta secretions from mice with a PE-like pathology may promote liver dysfunction including excessive SAA production. Increasing hepatic SAA may be one of the contributing factors in triggering maternal kidney inflammation during pregnancy. Therefore, we suggest that there is an in vivo organ interaction between the placenta, liver, and kidney during pregnancy, and functional abnormalities of each organ may be exacerbated of pathogenesis of PE. Understanding these mechanisms may help elucidate the pathogenesis of PE and the development of novel therapeutic strategies for PE.

## 4. Materials and Methods

### 4.1. Animal Experiments

All experiments were approved by the Ethics Committee on Animal Rights Protection and conducted following the Tokyo University of Agriculture Guide for Laboratory Animals. Female ICR mice (8–10 weeks old, purchased from Japan SLC Inc., Shizuoka, Japan) were mated with male ICR mice. The presence of a vaginal plug was checked the following morning, which was designated as GD1. The blood pressure of the mice was measured using the tail cuff method in all experiments, as previously described [13].

In vivo experiment 1: Ang II infusion was performed as previously described [13]. In brief, pregnant mice were infused with saline (*n* = 10) or Ang II (*n* = 11, 670 ng/kg/min; A9525, Sigma-Aldrich, St. Louis, MO, USA) on GD12 using an osmotic mini pump (Alzet osmotic pumps, Alzet, Campbell, CA, USA). Ang II is dissolved in filtered ultrapure water and diluted with saline (Otsuka Pharmaceutical Co., Ltd., Tokyo, Japan) before administration.

All mice were sacrificed on GD18, and plasma, placenta, fetus, liver, and kidneys were collected. The placenta (*n* = 4–6 in each experiment, with 3–4 placental tissue were cultured in each dam) was cultured in Dulbecco’s Modified Eagle Medium/F-12 (DMEM/F-12, Thermo Fisher Scientific, Waltham, MA, USA) supplemented with 5% fetal calf serum (FCS, ICN Pharmaceuticals, Inc., Costa Mesa, CA, USA) and antibiotics (amphotericin B and gentamycin, Sigma-Aldrich). The CM was collected after 24 h of placental tissue culturing.

In vivo experiment 2: Pregnant mice were injected with saline (*n* = 5) or recombinant SAA (*n* = 6, 5 µg SAA/100 µL saline, CLOUD-CLONE CORP, Katy, TX, USA) through the tale vein every 2 days (GD12, 14 and 16). All mice were sacrificed on GD17, and plasma and tissues were collected.

### 4.2. Cell Culture and Experiments

NCTC clone 1469, mouse liver cells and TKC2, mouse tubule cells were purchased from JCRB cell bank [47,48] (Osaka, Japan). NCTC clone 1469 was cultured in DMEM/F-12 supplemented with 5% FCS and antibiotics. TKC2 cells were cultured with DMEM/F-12 supplemented with 2% FCS, epidermal growth factor (Fujifilm Wako Pure Chemical Corporation, Osaka, Japan), ITS-G Supplement (Fujifilm Wako Pure Chemical Corporation), and antibiotics. NCTC clone 1469 and TKC2 cells were seeded at densities of 2 × 10^5^ cell/mL in a 48-well culture plate. These cells were treated with placental-CM (10% addition of placental-CM), Ang II (0.1, 1, 10 µM), or SAA (2 µg/mL) for 24 h. CLI-095 (TLR4 inhibitor, 1 µM, InvivoGen, San Diego, CA, USA) was pre-treated for 1 h prior to SAA treatment.

### 4.3. Measurements of SAA and ALT

The levels of SAA were measured using a mouse ELISA kit (R&D Systems, Minneapolis, MN, USA) according to the kit instructions. The levels of ALT were measured using a commercial kit (BioVision Inc., Milpitas, CA, USA) according to the kit instructions. Absorbance was measured at 450 nm for SAA or 570 nm for ALT using a microplate spectrophotometer (DS Pharma Biomedical, Osaka, Japan).

### 4.4. RNA Sequencing (RNA-Seq)

Total RNA was extracted and mixed from maternal kidneys or liver (Control; *n* = 10, Ang II; *n* = 11), and RNA-seq analysis was performed similarly to the method described in our previous study [49]. RNA-seq was performed by BGI (Hyogo, Japan). After assessing the RNA quality (RNA Integrity Number [RIN] value ≥ 7.0) using a 2100 Bioanalyzer (Agilent Technologies, Santa Clara, CA, USA), DNA nanoball (DNB) SEQ standard mRNA sequencing libraries were prepared. Clean reads were then mapped onto the reference genome (Mus_musculus_ GCF_000001635.26_GRCm38.p6) using hierarchical indexing for the spliced alignment of transcripts. Differentially expressed genes (DEGs) between groups were identified using DESeq. Clustering analysis of DEGs and GO enrichment analysis were carried out for the signaling pathway annotation of DEGs.

### 4.5. Real-Time Reverse Transcriptase Polymerase Chain Reaction (RT-PCR)

RT-qPCR was performed as previously described [13]. In brief, RT-qPCR was performed using the CFX Connect™ Real Time PCR (Bio-Rad, Hercules, CA, USA) and a commercial kit (Thunderbird SYBR qPCR Mix; Toyobo Co., Ltd., Tokyo, Japan). The primers are listed in Supplemental Table S5. Expression levels were normalized to *18S rRNA* or *Gapdh* using the ^ΔΔ^ CT method.

### 4.6. Histology

Kidneys were randomly selected (*n* = 5 in each group) and fixed in 4% paraformaldehyde and embedded in paraffin. Tissue sections (4 µm thick) were subjected to PAS staining. Tubular injury was assessed using PAS staining in terms of necrotic lysis, tubular dilatation, tubular brush border loss, cast formation, and sloughing of cellular debris into the tubular lumen. The injury score was determined by the same nephrologist as described previously [50,51] according to the following grades: normal, grade 0; <25%, grade 1; 25–49%, grade 2; 50–74%, grade 3; and ≥75%, grade 4.

Immunohistochemistry (IHC) of paraffin-embedded sections was performed to evaluate CD45 and KIM-1 expression levels in the kidneys. Kidney sections were deparaffinized, boiled in Target Retrieval Solution (Dako, Carpinteria, CA, USA), blocked with 2% normal goat serum, and incubated with primary antibodies overnight: CD45 (#550539, BD Biosciences, Franklin Lakes, NJ, USA) and Mouse TIM-1/KIM-1/HAVCR (#AF1817, R&D Systems). Sections were incubated with Histofine Simple Stain MAX PO (Nichirei Bioscience, Tokyo, Japan), followed by DAB substrate kit (Vector Laboratories, Newark, CA, USA). All sections were counterstained with hematoxylin. Species-matched immunoglobulin G (IgG) was used as the negative control for primary antibodies. PAS- or IHC-stained sections were digitized using a microscope (APX100; Evident, Tokyo, Japan). IHC-positive areas were quantified using Image J software (ver. 2.16.0; National Institutes of Health).

For fluorescent staining, Alexa 647-conjugated Wheat Germ Agglutinin (Invitrogen, Waltham, MA, USA) and DAPI (Dojindo, Kumamoto, Japan) were used for the cell membrane and nuclei, respectively. Images of the stained sections were digitized using an Andor Dragonfly 200 spinning disk confocal unit (Andor, Belfast, UK) equipped with an IX83 microscope (Evident, Tokyo, Japan) and analyzed using Image J software.

### 4.7. Statistics

Data are expressed as the mean ± standard error of the mean (SEM). Differences between the treatment groups were identified using an unpaired *t*-test or Mann–Whitney U test. Multiple comparisons were examined via parametric analysis using one-way analysis of variance (ANOVA) with Tukey–Kramer multiple comparison or Welch one-way ANOVA with Bonferroni multiple comparison, and non-parametric analysis using the Kruskal–Wallis test with the Steel–Dwass comparison test (statistical software version 4.08, BellCurve, Social Survey Research Information, Tokyo, Japan). Statistical significance was set at *p* < 0.05.

## Data Availability

The original contributions presented in this study are included in the article/Supplementary Materials. Further inquiries can be directed to the corresponding author.

## Supplementary Materials

The following supporting information can be downloaded at: https://www.mdpi.com/article/10.3390/ijms262110737/s1.

## Abbreviations

The following abbreviations are used in this manuscript:

PE	Preeclampsia
Ang II	Angiotensin II
GD	Gestational day
SAA	Serum amyloid A
CM	Conditioned medium
ALT	Alanine aminotransferase
sFlt-1	Soluble fms-like tyrosine kinase-1
SBP	Systolic blood pressure
DBP	Diastolic blood pressure
IL-6	Interleukin-6
IL-1β	Interleukin-1β
