# Supplementary material for: Placenta-Derived Secretions Promote Liver Dysfunction, and Hepatic Serum Amyloid A Mediates Kidney Inflammatory Response in a Preeclampsia-like Mouse Model
Source: Int J Mol Sci. 2025 Nov 4;26(21):10737. doi: 10.3390/ijms262110737 (PMC12608534; doi:10.3390/ijms262110737)
Supplement: Supplementary file 1 [file ijms-26-10737-s001.zip › IJMS Ozawa Sup Table S5.pdf]

Table S5. Primer list.

Saa1	Fwd	CATTTGTTCACGAGGCTTTCC
	Rev	GTTTTTCCAGTTAGCTTCCTTCATGT
Saa2	Fwd	TGTGTATCCCACAAGGTTTCAGA
	Rev	TTATTACCCTCTCCTCCTCAAGCA
Lcn2	Fwd	GAAATATGCACAGGTATCCTC
	Rev	GTAATTTTGAAGTATTGCTTGTTT
Kim-1	Fwd	CTGGAATGGCACTGTGACATCC
	Rev	GCAGATGCCAACATAGAAGCCC
Cd45	Fwd	ATGGTCCTCTGAATAAAGCCCA
	Rev	TCAGCACTATTGGTAGGCTCC
Ccl2	Fwd	GGCTCAGCCAGATGCAGTTAAC
	Rev	GCCTACTCATTGGGATCATCTTG
Gapdh	Fwd	TGTGTCCGTCGTGGATCTGA
	Rev	TTGCTGTTGAAGTCGCAGGAG
18sRNA	Fwd	GGACCAGAGCGAAAGCATTTGCC
	Rev	TCAATCTCGGGTGGCTGAACGC
