# Supplementary figures and images for: Placenta-Derived Secretions Promote Liver Dysfunction, and Hepatic Serum Amyloid A Mediates Kidney Inflammatory Response in a Preeclampsia-like Mouse Model
Source: Int J Mol Sci. 2025 Nov 4;26(21):10737. doi: 10.3390/ijms262110737 (PMC12608534; doi:10.3390/ijms262110737)

Supplemental Figure S1

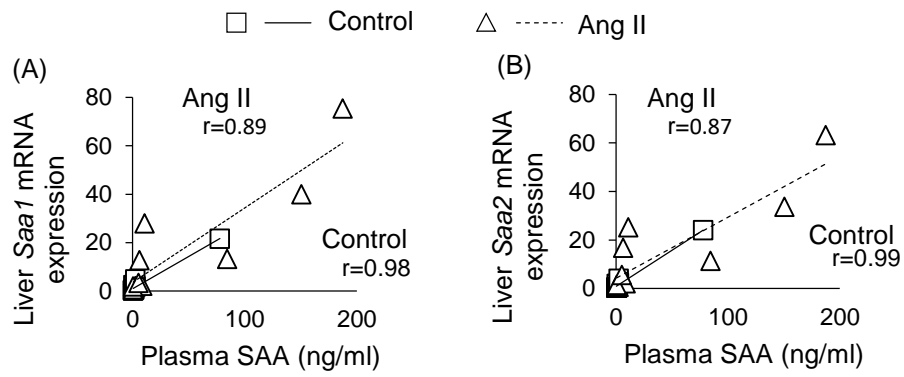

Supplemental Figure S2

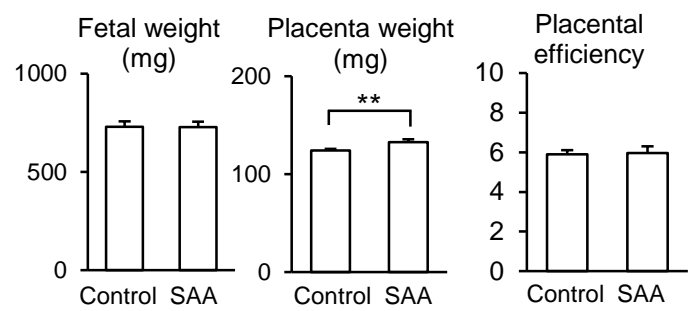

Supplement: Supplementary file 1 [file ijms-26-10737-s001.zip › Figures S1 and S2]
